# Translocation of *Zymomonas mobilis* pyruvate decarboxylase to periplasmic compartment for production of acetaldehyde outside the cytosol

**DOI:** 10.1002/mbo3.809

**Published:** 2019-02-15

**Authors:** Elina Balodite, Inese Strazdina, Jekaterina Martynova, Nina Galinina, Reinis Rutkis, Zane Lasa, Uldis Kalnenieks

**Affiliations:** ^1^ Institute of Microbiology and Biotechnology University of Latvia Riga Latvia

**Keywords:** acetaldehyde production, periplasm, pyruvate decarboxylase, *Zymomonas mobilis*

## Abstract

Acetaldehyde, a valuable commodity chemical, is a volatile inhibitory byproduct of aerobic fermentation in *Zymomonas mobilis* and in several other microorganisms. Attempting to improve acetaldehyde production by minimizing its contact with the cell interior and facilitating its removal from the culture, we engineered a *Z. mobilis* strain with acetaldehyde synthesis reaction localized in periplasm. For that, the pyruvate decarboxylase (PDC) was transferred from the cell interior to the periplasmic compartment. This was achieved by the construction of a *Z. mobilis* Zm6 PDC‐deficient mutant, fusion of PDC with the periplasmic signal sequence of *Z. mobilis* gluconolactonase, and the following expression of this fusion protein in the PDC‐deficient mutant. The obtained recombinant strain PeriAc, with most of its PDC localized in periplasm, showed a twofold higher acetaldehyde yield, than the parent strain, and will be used for further improvement by directed evolution.

## 1 INTRODUCTION

Acetaldehyde is a highly desirable product of microbial biosynthesis, because it can be further used as the entry point for the synthesis of acetic acid, acetic anhydride, butadiene, crotonaldehyde, and other higher value chemicals (Danner & Braun, 1999; Moore et al., 2017). Several microorganisms, like *Zymomonas mobilis*, genetically engineered *Lactococcus lactis*, or yeast, have been investigated for the production of acetaldehyde from sugary substrates (Bongers, Hoefnagel, & Kleerebezem, 2005; Wecker & Zall, 1987). Acetaldehyde is the direct metabolic precursor of ethanol. It accumulates in aerobic culture as a volatile byproduct, due to withdrawal of reducing equivalents from the ADH reaction by respiration. The advantage of *Z. mobilis* as an acetaldehyde producer is the best rate of its Entner–Doudoroff glycolytic pathway, a very active pyruvate decarboxylase, and, at the same time, an active respiratory chain with low energy‐coupling efficiency, ideally suited for regeneration of NAD^+^ under condition when ethanol is not the major catabolic product (Kalnenieks, 2006; Rogers, Jeon, Lee, & Lawford, 2007). So far, only 40%–50% of the theoretical yield have been reached in acetaldehyde bioprocesses, hence a considerable potential for yield improvement remains. Synthesis of ethanol and other byproducts from acetaldehyde prevents reaching the maximum yield, which is 2 moles of acetaldehyde per mole of catabolized glucose.

**Figure 1:**
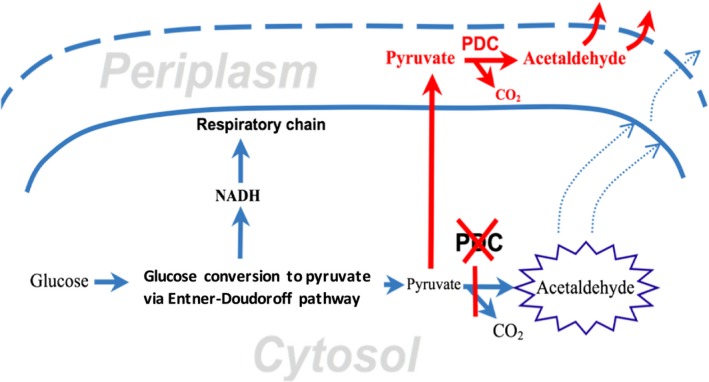
Acetaldehyde production in *Zymomonas mobilis* cell. Metabolic modifications are shown in red

Acetaldehyde inhibits microbial growth already at millimolar concentrations (Wecker & Zall, 1987). At first glance, it should be possible to develop resistant producer strains in a straightforward manner, by means of directed evolution in the presence of externally added acetaldehyde. However, since acetaldehyde is being produced in the cytoplasm, some of the stress protection mechanisms thus attained, like lowering of membrane permeability for acetaldehyde or binding and degrading of its intracellular pool (Aranda & del Olmo, 2004; Matsufuji et al., 2008), would decrease the overall productivity rather than contributing to it. More likely, further progress here depends on improved methods for acetaldehyde removal both from the cell interior and from the culture medium. Acetaldehyde has no specific membrane permease. Study with yeast (Stanley & Pamment, 1993) suggests that its diffusion through the lipid bilayer is slower than that of ethanol, and may represent a serious bottleneck for acetaldehyde producers. The aim of the present study was to circumvent this bottleneck, by moving the acetaldehyde‐generating reaction (pyruvate decarboxylase; PDC) from the cell interior to the periplasmic compartment (Figure 1). Acetaldehyde, when generated in the periplasm and removed from the culture medium by gassing, can be expected to cause less damage to the cell interior, and also, to be less accessible for the cytosolic enzymes, converting it into ethanol or acetate. That could in future serve as a valid basis for developing acetaldehyde producer strains with both improved yield and increased acetaldehyde resistance. Here we report relocation of acetaldehyde synthesis from cytosol to periplasm by three steps: (a) construction of *Z. mobilis* Zm6 PDC‐deficient mutant, (b) construction of a PDC fusion with the 35 amino acid periplasmic signal sequence of *Z. mobilis* gluconolactonase (Kanagasundaram & Scopes, 1992), and (c) its expression in the PDC‐deficient mutant.

## 2 RESULTS AND DISCUSSION

### 2.1 Construction of a PDC‐deficient strain and expression of PDC with a periplasmic signal sequence

PDC in the wild type *Z. mobilis* is an abundant and highly active enzyme; hence almost all pyruvate coming from the E‐D pathway gets decarboxylated in the cytosol. Therefore, we regarded a PDC‐deficient strain as a more suitable genetic background for constructing the pathway of periplasmic acetaldehyde production, than the wild type. Cytosolic PDC deficiency could be expected to stimulate pyruvate export, thus providing the periplasmic PDC with substrate

**Figure 2:**
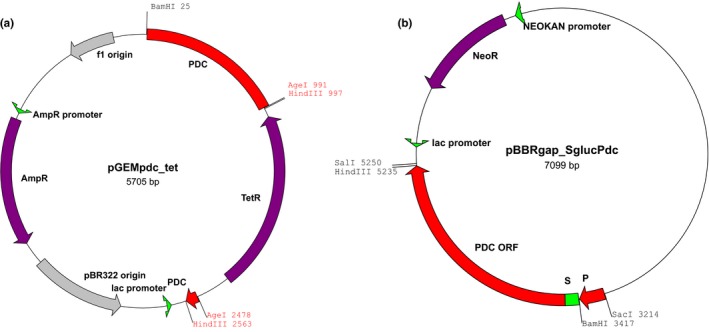
Plasmids used in the study. (a) pGEMpdc_tet. Construction: (i) 1.58 kb fragment of the *pdc* ORF was amplified with the primer pair Pdc1 and Pdc2, double‐digested with *Bam*HI and *Hin*dIII, and cloned between the respective sites in the vector pGEM3Zf(+); (ii) the resulting construct was digested with *Age*I, cutting out an 0.53 kb fragment from the *pdc *ORF; (iii) a 1.49 kb DNA fragment, carrying tetracycline resistance determinant (tet^r^) was amplified with primers TetAge1 and TetAge2, taking plasmid pBR322 for template, digested with *Age*I, and inserted between the *Age*I sites of the cloned gene. (b) pBBRgap_SglucPdc. Construction: (i) the promoter region (P) of the glyceraldehyde‐3‐phosphate dehydrogenase gene (*Z. mobilis* Zm6 genome sequence, locus tag ZZ6_RS05365) was amplified with the primer pair Gap1 and Gap2, and the resulting 0.2 kb product was double‐digested with *Sac*I and *Bam*HI, and cloned between the respective sites in the vector pBBR1MCS‐2; (ii) 1.8 kb PCR product obtained with the primer pair SglucPdc_fwd and Pdc_rev was double‐digested with *Bam*HI and *Hin*dIII, and cloned next to P. All constructs were cloned and maintained in *E. coli* JM109

For construction of the PDC‐deficient strain (Pdc‐) we chose the same approach, previously proved efficient for construction of other *Z. mobilis* knock‐out mutants (Kalnenieks et al., 2008; Strazdina et al., 2012). A construct on the basis of plasmid vector pGEM3Zf(+) (Figure 2a), carrying major part of *pdc *ORF with an inserted tetracycline resistance marker and unable to propagate in *Z. mobilis*, was used to transform the wild type strain *Z. mobilis* Zm6 (ATCC 29,191). Colonies of homologous recombinants were selected on plates containing tetracycline (20 g/ml), and verified by PCR with the primer pair Pdc1 and Pdc2 (Table 1), taking genomic DNA as the template

**Table 1:** Primers used in the study

Primer		Sequence		Restriction site (underlined)	Notes
Pdc1		gtccagattggatccaagcatcacttcgcag		*Bam*HI	
Pdc2		cttcagtgcaagcttcacgaccgatgaagc		*Hin*dIII	
TetAge1		cagcttatcaccggtaagctttaatgcgg		*Age*I	
TetAge2		gattcattctgctaaccggtaaggcaaccc		*Age*I	
Gap1		gacaatgagctcggaacggtatactg		*Sac*I	
Gap2		caactttaaccgccatggatcctctc		*Bam*HI	
SglucPdc_fwd		agaggatccATGACCACTGGTCGTATGTCTCGTCGAGAATGCCTTTCCGCAGCTGCCATAGTGCCTATTGCCGCTATGACAGCCACCGCCACTATTACAGGATCAGCTCAGGCTatgagttatactgtcggtac		*Bam*HI	105 bp fragment encoding the 35 aa signal sequence of gluconolactonase shown in capital letters
Pdc_rev		gtttattaagcttctagaggagc		*Hin*dIII	

For expression of periplasmic pyruvate decarboxylase, carrying the 35 amino acid periplasmic signal sequence of *Z. mobilis* gluconolactonase (EC 3.1.1.17; UniProtKB accession Nr. Q 01578) attached to its *N*‐end, a 134 bp primer SglucPdc_fwd was synthesized. It encoded the signal sequence (S; Figure 2b), fused to the initial part of the *pdc* ORF. Plasmid construct pBBRgap_SglucPdc (Figure 2b) was built on the basis of shuttle vector pBBR1MCS‐2, and carried the PCR product of the primer pair SglucPdc_fwd and Pdc_rev (Table 1) under the glyceraldehyde‐3‐phosphate dehydrogenase gene promoter (P; Figure 2b). The plasmid construct was used for transformation of the strain Pdc‐. Transformation with the pBBR construct and selection of transformants (strain *Z. mobilis* PeriAc) was done, following the routines described previously (Balodite et al., 2014).

**Figure 3:**
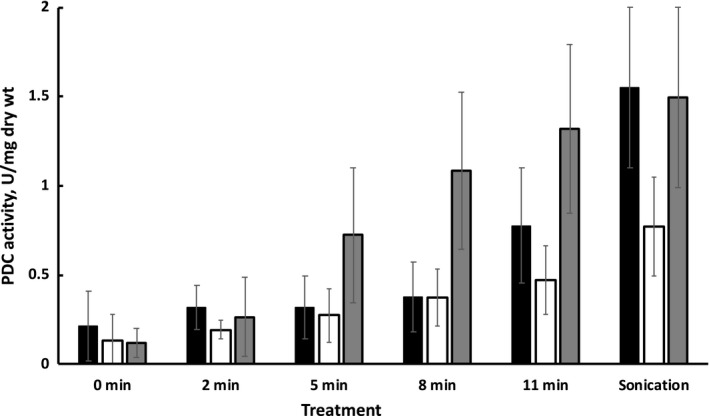
PDC activity in cell‐free extracts and in extracellular medium after lysozyme treatment of various duration: Zm6 (black columns), Pdc‐ (empty columns), and PeriAc (gray columns). Assay for lysozyme treatment: 100 L of 100 mg/ml lysozyme was added to 900 L of 4 mg/ml washed cell suspension (harvested at mid‐exponential phase of aerobic growth) in 0.2 M TRIS/HCl buffer, pH 8, and incubated at room temperature by vortexing of various duration. Then, samples were rapidly cooled in an ice bath, centrifuged, and the supernatants used for PDC activity measurement. Cell‐free extracts were prepared by 2 min ultrasonic disruption with pulses of 0.5 s duration, separated by 0.5 s intervals. PDC activity assay: 5 L of the supernatant from lysozyme‐treated or sonicated cell suspension was added to a mixture, containing 937 L of 0.2 M citrate buffer, pH 6, 38 L of 1 M pyruvate, 15 L of 10 mM NADH, and 5 L of ADH. Results represent mean values of five experiments, with error bars showing standard deviation

The PDC‐deficient strains appeared to be “leaky,” very much like those, recently derived from Zm4 by Zhao, Rogers, Kwon, Jeong, and Jeon (2015), using methodology similar to ours. In spite of the fact that both Zm4 and Zm6 genome sequences contain one copy of the pyruvate decarboxylase gene, in the recombinants PCR reaction on chromosomal DNA template amplified two products, one of which was identified by sequencing as the disrupted gene copy with the antibiotic resistance insert, and the other one being the intact gene copy. In our mutant strain *Z. mobilis* Pdc‐, the primer pair Pdc1 and Pdc2 amplified a 1.6 kb fragment, corresponding to the intact gene, and a longer fragment of about 2.5 kb, carrying the tet^r^ insert. Both were seen on agarose gel as two bands of comparable intensity (not shown). The mechanism of the apparent PDC gene duplication in these experiments still needs to be clarified, but possibly, such phenomenon is somehow related to the essentiality of PDC for catabolism and ethanologenesis in *Zymomonas*. Nevertheless, the Pdc‐ strain showed a partially PDC‐deficient phenotype: in the cytosol fraction of cell‐free extracts, prepared by ultrasonic breakdown and removal of membranes by centrifugation (as described in Strazdina et al., 2012) the mutant's PDC activity constituted half of that seen in the wild type Zm6 (Figure 3). A similar 50% decrease of PDC activity was reported also by Zhao et al. (2015).

### 2.2 Intracellular location of PDC

In order to monitor the distribution of PDC between cytosol and periplasm in the parent strain and both mutants, cells were incubated in lysozyme‐containing TRIS/HCl buffer for various time spans to disrupt the external membrane, basically following the procedure of Linger, Adney, and Darzins (2010). The PDC activity was then measured in the extracellular medium. As seen in Figure 3, there was a background activity of PDC in the incubation medium before lysozyme treatment in all three strains, presumably due to some cell lysis happening during the pretreatment (cell sedimentation, washing, and concentration steps). Incubation with lysozyme for 2 min practically did not add to this activity. Incubation for 5 min, and, especially for 8 min, raised the medium PDC activity in PeriAc, but still no significant change was seen in both other strains. Incubation for 11 min, however, produced a increased PDC activity in the extracellular medium in both PeriAc and Zm6, and to a lesser extent in Pdc‐. Given that in the wild type PDC is localized in cytosol, detection of its activity after 11 min of lysozyme treatment indicated disruption of the cytoplasmic membrane. Therefore, we concluded that the 8 min interval was close to the optimum length for lysozyme treatment, releasing the periplasmic pool of PDC in the strain PeriAc, while leaving intact the cytosolic compartment. Comparison of the PDC activity in PeriAc cell‐free extract (sonicate) with the extracellular activity after 8 min lysozyme treatment indicates that more than half of PDC in this strain is periplasmic. Notably, in cell‐free extracts the total activities of PDC in wild type and PeriAc were closely similar, so these strains differed mainly by their PDC localization.

### 2.3 Growth and product synthesis in the PDC mutant strains

**Figure 4:**
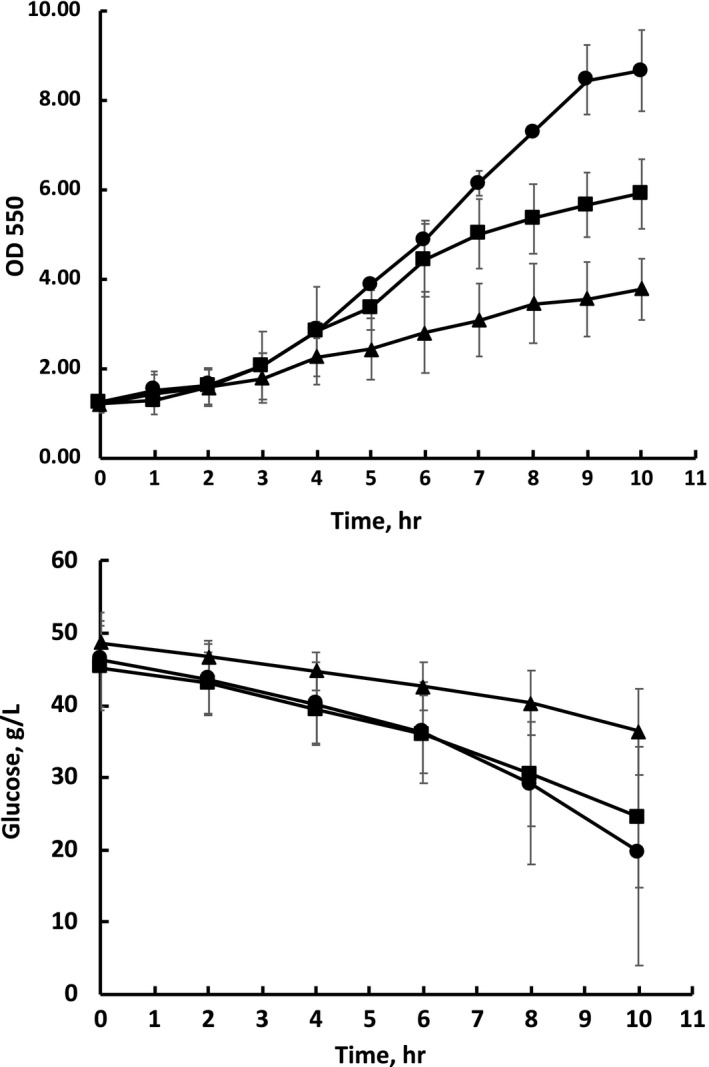
Growth and glucose consumption of aerobic batch cultures. Circles—Zm6, squares—Pdc‐, triangles – PeriAc. Cultivation took place in 1 L unbaffled flasks with 160 ml of culture on a rotary shaker at 155 r.p.m. Glucose concentration was measured by HPLC (Strazdina et al., 2012). Results represent mean values of three experiments, with error bars showing standard deviation

**Figure 5:**
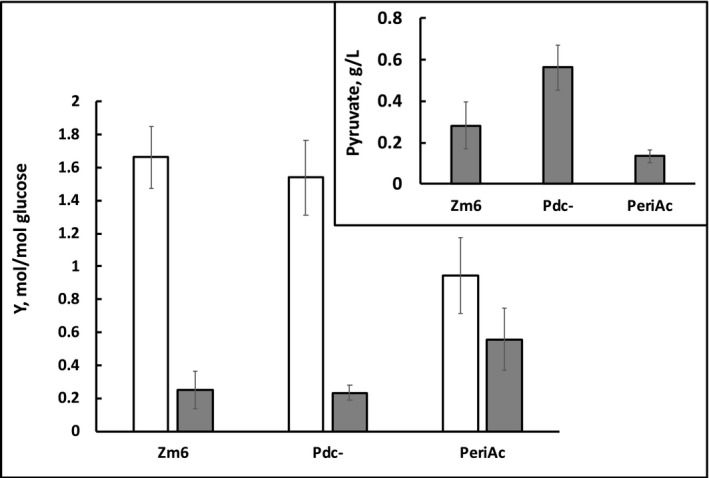
Product yields after 10 hr of aerobic growth. Empty columns—ethanol, filled columns—acetaldehyde; grams produced per gram of glucose. Inset: pyruvate accumulated in the medium. Ethanol and glucose concentrations were measured by HPLC (Strazdina et al., 2012). Acetaldehyde concentration was measured with Megazyme acetaldehyde kit K‐ACHYD, following manufacturer's instructions. For pyruvate determination, 100 L of the sample was added to assay mixture containing 500 μL of 0,1 M TEA buffer, pH 7.6, 375 μL of 74 mM KCl +20 mM MgCl_2_, and 15 μL of 6 mM NADH. Results represent mean values of four experiments, with error bars showing standard deviation

**Figure 6:**
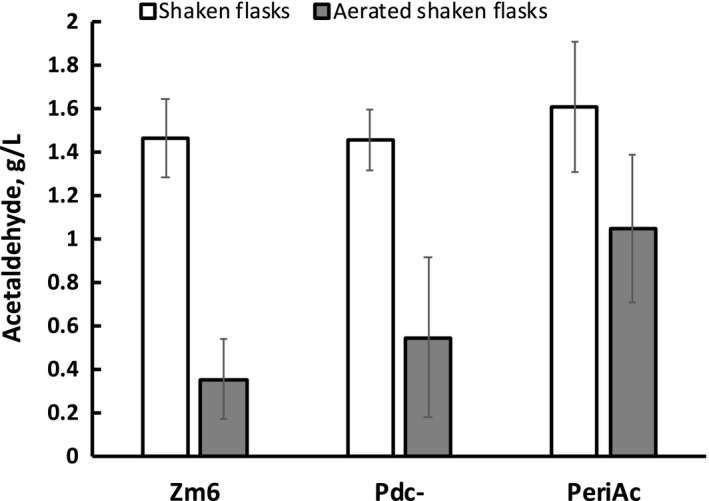
Acetaldehyde concentration in media after 10 hr of aerobic cultivation. Empty columns—shaken flasks, filled columns—shaken flasks with gassing of air

Periodical cultivations were done aerobically in shaken flasks, since respiration in mutants is essential for regeneration of NAD+ when the overall redox balance in ethanologenesis is compromised by export of pyruvate. The Pdc‐ strain in shaken flasks grew slower and reached lower final biomass concentration, than the parent strain (Figure 4). The specific growth rate, calculated for the period between the second and sixth hour after inoculation, was 0.28 hr^−1^ for Zm6 and 0.25 hr^−1^ for Pdc‐. Specific rates of glucose consumption for both strains were closely similar, 16.5 mmol (g_dry wt_ **^.^**hr)^−1^ and 17.6 mmol (g_dry wt_ **^.^**hr)^−1^, respectively. PDC‐deficiency caused a slight decrease in ethanol yield, and more pyruvate was accumulated in the medium by the early stationary phase, than in both other strains (Figure 5). However, the strain PeriAc showed even lower specific growth and glucose consumption rates (0.15 hr^−1^ and 13.5 mmol (g_dry wt_ **^.^**hr)^−1^, respectively). It had a considerably lower ethanol yield, but at the same time, at least a twofold higher yield of acetaldehyde (this estimation is based on acetaldehyde concentration in the culture medium, not taking into account evaporation), than both other strains under the given culture conditions. Differences in the ethanol and acetaldehyde yields between PeriAc and Pdc‐ were found to be statistically significant (*p *< 0.05). Accordingly, PeriAc accumulated the least amount of pyruvate in the medium (Figure 5, inset), but the concentration of acetaldehyde in its culture medium, reached after 10 hrs of cultivation, was 1.61  0.3 g L^−1^, slightly exceeding the average values of Zm6 (1.47  0.18 g L^−1^) and Pdc‐ (1.46  0.14 g L^−1^), although the differences were not statistically significant (Figure 6, empty bars). The relatively slower growth of PeriAc thus hardly can be explained by excessive accumulation of acetaldehyde.

Such metabolic properties of PeriAc seem to be in a good agreement with the fact that most of PDC is located outside the cytosol. When exported from the cytosol of PeriAc, pyruvate gets captured and decarboxylated to acetaldehyde in the periplasm, so accordingly, a smaller its fraction reaches external medium, than in the case of Pdc‐. Decarboxylation of pyruvate could be expected to maintain a higher pyruvate concentration gradient between the cell interior and periplasm, and thus to stimulate pyruvate export from the cytosol. Acetaldehyde, generated in the periplasm, from the very beginning is spatially separated from the alcohol dehydrogenases by the cytoplasmic membrane barrier. Hence, there is a higher probability for it to leave the cell via the outer membrane, than to interact with the cytosolic alcohol dehydrogenases, in contrast to both other strains, in which all acetaldehyde is generated in the cytosol.

It is important to realise, however, that if we let acetaldehyde to accumulate in the growth medium, then after some time it would anyway penetrate the cytosol of PeriAc, and the recombinant strain would perform like the wild type. Therefore, to take advantage of the periplasmic localization of PDC, efficient removal of acetaldehyde from the extracellular medium is mandatory. Gassing the shaken cultures with air at the flow rate of 2 vol vol^−1^min^−1^ largely facilitated removal of acetaldehyde from the culture media (Figure 6). Notably, relative to the shaken flasks without gassing, a much bigger difference between the acetaldehyde levels in PeriAc and in both other strains was established (Figure 6; filled bars). In PeriAc, the removal of acetaldehyde appeared to stimulate its synthesis more than in other strains, since by the end of cultivation the quasi steady‐state acetaldehyde concentration in PeriAc culture medium was almost three times higher than in Zm6. As expected, by decreasing acetaldehyde concentration, gassing stimulated growth. After 12 hrs with gassing, the optical density of Zm6 and Pdc‐ reached twice the control values, but in PeriAc it was higher by 60% (not shown).

PeriAc provides a promising basis for development of acetaldehyde producers of a novel type. Further research is needed to characterize its growth and production at various aeration intensities, and to ensure efficient product recovery. Also, directed evolution in aerobic chemostat, gradually raising the flow rate, as well as selective pressure by externally added acetaldehyde, would help to improve the strain performance—its growth and production rates, and acetaldehyde resistance.

## CONFLICT OF INTEREST

The authors declare no conflict of interest.

## AUTHORS CONTRIBUTION

UK, EB, and IS designed the research; EB, IS, JM, NG, RR, and ZL performed the experiments; IS, EB, NG, and UK analyzed the data; UK wrote the paper. All the authors have approved the manuscript.

## ETHICS STATEMENT

None required.

## DATA ACCESSIBILITY

All data are provided in full in the results section of this article. Strains Pdc‐ and PerAc are deposited in the Microbial strain collection of the University of Latvia and are made available on request.
